# Delays in Obtaining Abortion and Miscarriage Care Among Pregnant Persons in New York State During the COVID-19 Pandemic: The CAP Study

**DOI:** 10.1089/whr.2023.0128

**Published:** 2024-01-17

**Authors:** Sarah Pickering, Meredith Manze, Jessie Losch, Diana Romero

**Affiliations:** Department of Community Health and Social Sciences, City University of New York (CUNY) Graduate School of Public Health and Health Policy, New York, New York, USA.

**Keywords:** abortion, reproductive health, miscarriage, COVID-19

## Abstract

**Background:**

We sought to investigate delays obtaining abortion and miscarriage care during the COVID-19 pandemic, compared with before the pandemic, among pregnant persons in New York State (NYS).

**Methods:**

We administered a cross-sectional survey in June–July 2020 to NYS residents aged 18–44 years who identified as female or transgender male (*N* = 1,525). This analysis focused on a subsample who had an abortion or miscarriage during COVID-19, were seeking an abortion at the time of the survey, or had an abortion or miscarriage before COVID-19 (*n* = 116). We conducted bivariate analyses to determine differences in delays to seeking or obtaining an abortion or miscarriage during versus before the pandemic, as well as consideration of abortion among those pregnant during versus before the pandemic. We also asked open-ended questions about miscarriage and abortion experiences.

**Main Findings:**

Of the 21 respondents who sought or were seeking an abortion during the COVID-19 pandemic, 76.2% (*n* = 16) reported experiencing a delay in obtaining abortion care, compared with 18.2% (*n* = 4) of those who experienced a delay before the pandemic (*p* < 0.001). A significantly higher proportion of respondents who were pregnant during the pandemic considered abortion, compared with those who gave birth before the pandemic (39.1% vs. 7.6%; *p* < 0.001). Of the 39 respondents who miscarried during the pandemic, 35.9% (*n* = 14) delayed care, compared with 5.9% (*n* = 2) before the pandemic (*p* < 0.01). Some respondents also commented on the difficulty of accessing miscarriage services during COVID-19 in open-ended responses.

**Principal Conclusions:**

Those who sought abortion or miscarriage care during the COVID-19 pandemic experienced significant delays in getting care. These are essential services that must be available during public health emergencies, and yet access to these services is now severely limited in many states due to the Dobbs vs. Jackson Women's Health Organization decision.

## Introduction

The COVID-19 pandemic significantly disrupted access to health care in the United States.^1,2^ Studies have shown delays specifically in accessing general sexual and reproductive health (SRH) services due to the pandemic.^3^ For example, in a study by the Guttmacher Institute, one-third of surveyed women reported experiencing delays or cancellations of contraceptive or other SRH care due to COVID-19.^4,5^ It is important to understand the impact of the pandemic on access to abortion and miscarriage care, which has been further disrupted by the *Dobbs vs. Jackson Women's Health Organization* decision that took place in June 2022, ending the constitutional right to abortion.^6^

The COVID-19 pandemic had a documented impact on access to abortion, with clinics across the country reporting reduced provider coverage and having to cancel or postpone abortion appointments.^7^ Despite the well-documented demand for increased accessibility,^8,9^ some state governors and legislatures attempted to limit access to abortion during the pandemic by categorizing abortion as a “nonessential” service that can be delayed or postponed.^10^ Many public health professionals cited the COVID-19 pandemic as a new and pressing reason to end restrictions and make abortion more accessible, not less so.^11^ However, states enacted more than 100 abortion restrictions in 2021 alone, the most in any year since the federal legalization of abortion in 1973.^12^

Since the *Dobbs* decision, as of the writing on this article, almost all abortions are banned in 14 states.^13^ Lack of access to abortion is associated with poor health outcomes for both pregnant people^14–16^ and infants, and yet states with the most abortion restrictions tend to have the fewest resources dedicated to women and children and the worst maternal and neonatal health outcomes.^17,18^ The World Health Organization states that lack of access to safe, timely, affordable, and respectful abortion care is a critical public health and human rights issue.

Many restrictions on abortion impact miscarriage care as well, as some of the services and medications used for abortion are also used for miscarriage management.^19^ Miscarriage is defined as pregnancy loss before 20 weeks of gestation,^20^ and access to miscarriage care and support is an essential service.^21–23^ While qualitative research on miscarriage experiences during COVID-19 is limited, one study in the United Kingdom found through semistructured interviews that participants reported experiencing fragmented, impersonal health care and confusion about pandemic restrictions relating to their miscarriage management.^24^ Many news and media outlets reported the exacerbated difficulties of miscarrying during the pandemic, with people sharing increased concerns about the safety of getting health care services, accessing services without support people present, or being discouraged from accessing care by health care providers.^25–28^

We sought to document people's experiences with seeking or obtaining abortions and miscarriage care in New York State (NYS) during the pandemic. Such experiences are important for devising relevant policy and programmatic interventions to avert further delays or other barriers to care during future crises, including possible new illnesses and subsequent lockdowns. While people typically experience abortion and miscarriage very differently, we included both experiences in our analyses because the clinical needs of those seeking abortion or experiencing miscarriage are often similar.

## Materials and Methods

### Study design

We conducted an observational study on access to health care in the context of pregnancy. We developed and administered a web-based survey with a Qualtrics^®^ respondent panel from June 9 to July 21, 2020. Respondents (*N* = 1,525) identified as female (*n* = 1,523) or transgender male (*n* = 2), were aged 18 to 44, and living in NYS at the time of the survey. We enrolled a sample of individuals who had been pregnant before the pandemic, were pregnant during the pandemic, or had considered becoming pregnant sometime in 2020. Primary analysis compared experiences of respondents who were pregnant during the pandemic with those who were pregnant before the pandemic.^29,30^

For the purposes of this analysis, we focused on a subset of respondents who had either had an abortion or miscarriage during COVID-19,^31^ were seeking an abortion at the time of the survey, or had an abortion or miscarriage before COVID-19 (defined as May 1, 2018–April 30, 2019) (*n* = 116). For the comparison group of those pregnant before the COVID-19 pandemic, we enrolled those pregnant between May 2018 and April 2019; we chose this date range because we wanted to include people whose pregnancies did not overlap with those affected by the pandemic, without going further back in time. This allowed us to compare mutually exclusive groups. The study was approved by the City University of New York Institutional Review Board (Protocol No. 2020-0339).

### Study sample

We sampled from five regions in NYS: New York City (5 counties), upstate and rural New York (47 counties), upstate New York with urban centers (5 counties), Long Island (5 counties), and the Hudson Valley (5 counties). We also sampled by race/ethnicity and by age approximately proportional to the NYS population distribution.

### Survey data/measures

We asked all participants who sought or obtained an abortion: “Did you experience a delay getting abortion care?” For those who were pregnant during the pandemic, response options were as follows: (1) Yes, due to COVID-19 restrictions; (2) Yes, due to COVID-19 and other reasons; (3) Yes, due to reasons other than COVID-19; and (4) No. Those who were pregnant before the pandemic had the following response options: (1) Yes, due to… (please specify) and (2) No.

We also asked about the type of abortion obtained, the setting of the abortion, and if the abortion involved virtual contact with a health care provider. First, we asked, “Was the kind of abortion you received…” (1) an abortion procedure by a health care provider; (2) medication (pills) abortion prescribed by a health care provider; (3) through other means (specify). The next question was: “Did you obtain abortion services from a…” (1) clinic that specializes in abortion services (*e.g.*, Planned Parenthood, Whole Women's Health); (2) clinic that provides mostly other types of services; (3) private doctor's office; (4) hospital; (5) other (specify). Last, we asked, “Did the abortion service you received involve virtual (telemedicine) contact with a health care provider?” with response options of Yes, No, and Not sure.

For all respondents who were pregnant during or before the pandemic but did not seek an abortion, we asked if they considered having an abortion. For those who were pregnant during the pandemic, response options were as follows: (1) Yes, due to COVID-19; (2) Yes, due to COVID-19 and other reasons; (3) Yes, due to reasons other than COVID-19; and (4) No. For those who were pregnant before the pandemic, response options were as follows: “Yes, due to… (please specify)” and “No.”

We asked participants who experienced a miscarriage during COVID-19 if they delayed obtaining miscarriage care due to COVID-19, or if a health care provider delayed seeing them for miscarriage care due to COVID-19. The specific questions were as follows: “Did you delay seeking care once you suspected you may be having a miscarriage” and “was your health care provider delayed in being able to see you, once you suspected you may be having a miscarriage?” Answer choices were as follows: (1) Yes, due to COVID-19; (2) Yes, due to COVID-19 and other reasons; (3) Yes, due to reasons other than COVID-19; (4) No; and (5) I did not seek care. We also asked what health care services were available (*e.g.*, virtual appointments) for those who sought care for their miscarriage.

For those who experienced a miscarriage before the pandemic, we asked the same questions with the following response options: (1) Yes, due to… (specify); (2) No; and (3) I did not seek care.

All participants were given the opportunity to answer an open-ended question about their experience with abortion or miscarriage before or during COVID-19.

### Analysis

We compared the experiences of those who sought or obtained an abortion or miscarriage care during and before COVID-19.

We conducted bivariate analyses with chi-square or Fisher's exact tests to determine differences in variables between those who sought or obtained an abortion or miscarriage care during or before the pandemic, as well as consideration of abortion during pregnancy between those pregnant during or before the pandemic. We aggregated all “yes” responses regarding delaying abortion or miscarriage care for the group pregnant during the COVID-19 pandemic to compare their reports of delays with respondents who were pregnant before the pandemic. Those who had a miscarriage but did not seek care were combined with those who reported not experiencing a delay obtaining care. We ran descriptive statistics on the type of abortion, type of facility where the abortion was obtained, use of virtual contact for the abortion, and types of appointments for miscarriage care.

For the open-ended responses related to miscarriage experiences, two members (S.P. and J.L.) of the research team independently reviewed all open-ended responses, generated a list of categories, and applied the categories to summarize the qualitative data. The team convened to consider and reconcile any uncertain categorizations. Thematic analysis was conducted with the final categorized data. We did not analyze the open-ended responses relating to abortion experiences due to a low response rate.

## Results

Almost half of the 1,525 respondents had a pregnancy during the COVID-19 pandemic (*n* = 681, 44.7%). Descriptive statistics for age, race/ethnicity, region of residence, relationship status, education level, income, and health insurance are shown below (Table 1). Of those who were pregnant during the pandemic, 16 (2.3%) had an abortion, 5 (0.73%) were planning an abortion, 239 considered abortion (39.1%), and 39 (5.7%) had a miscarriage (Table 2). Almost one-fifth of study respondents (*n* = 296) had a pregnancy before the COVID-19 pandemic, during May 2018 to April 2019. Of those, 22 (7.4%) had an abortion, 18 (7.6%) considered abortion, and 34 (11.5%) had a miscarriage.

**Table 1.** Sample Characteristics

Variables	Total (***N*** = 373)	Sought/obtained abortion (***n*** = 43), ***n*** (%)	Considered abortion during pregnancy (***n*** = 257), ***n*** (%)	Experienced a miscarriage (***n*** = 73), ***n*** (%)
Age group				
18–24	155	22 (14.2)	108 (69.7)	25 (16.1)
25–34	135	14 (10.4)	97 (71.9)	24 (17.8)
35–44	83	7 (8.4)	52 (62.7)	24 (28.9)
Race/ethnicity				
White, non-Hispanic	193	22 (11.4)	129 (66.8)	42 (21.8)
Black, non-Hispanic	75	5 (6.7)	53 (70.7)	17 (22.7)
Hispanic, Latino	80	11 (13.8)	61 (76.3)	8 (10.0)
Asian	15	2 (13.3)	9 (60.0)	4 (26.7)
Other	10	3 (30.0)	5 (50.0)	2 (20.0)
New York State region of residence				
New York City	234	26 (11.1)	175 (74.8)	33 (14.1)
Long Island	16	3 (18.8)	6 (37.5)	7 (43.8)
Hudson Valley	13	2 (15.4)	7 (53.8)	4 (30.8)
Upstate—urban	43	7 (16.3)	25 (58.1)	11 (25.6)
Upstate—rural	67	5 (7.5)	44 (65.7)	18 (26.9)
Relationship status				
Married/cohabiting	259	18 (6.9)	199 (76.8)	42 (16.2)
Relationship/not cohabiting	43	11 (25.6)	20 (46.5)	12 (27.9)
Single/other	71	14 (19.7)	38 (53.5)	19 (26.8)
Highest educational level^a^				
Less than or equal to HS	100	8 (8.0)	71 (71.0)	21 (21.0)
Some college or AA	120	20 (16.7)	71 (59.2)	29 (24.2)
BA or more	152	15 (9.9)	114 (75.0)	23 (15.1)
Income, mean^b^				
$0–$28k	119	19 (16.0)	73 (61.3)	27 (22.7)
$28k–$60k	109	13 (11.9)	71 (65.1)	25 (22.9)
$60k+	143	11 (7.7)	112 (78.3)	20 (14.0)
Health insurance				
No insurance	13	4 (30.8)	4 (30.8)	5 (38.5)
Private/employer/exchange	91	18 (19.8)	47 (51.6)	26 (28.6)
Medicaid (regular)	140	15 (10.7)	98 (70.0)	27 (19.3)
Temporary Medicaid/other	129	6 (4.7)	108 (83.7)	15 (11.6)

^a^ One response missing.

^b^ Two responses missing.

**Table 2.** Abortion-Related Experiences of Those Pregnant Before the COVID-19 Pandemic Compared with Those Pregnant During the Pandemic

Variables	Total sample who had or sought an abortion (***N*** = 43), ***n*** (%)	Obtained abortion before COVID-19 (***n*** = 22), ***n*** (%)	Obtained (***n*** = 16) or sought an abortion during COVID-19 (***n*** = 5) (total ***n*** = 21), ***n*** (%)
Experienced a delay obtaining abortion?^a^			
Yes, due to COVID-19	11 (25.6)	—	11 (52.4)
Yes, due to COVID-19 plus other reasons	3 (7.0)	—	3 (14.3)
Yes, due to reasons other than COVID-19	6 (14.0)	4 (18.2)	2 (9.5)
No	23 (53.5)	18 (81.8)	5 (23.8)
Experienced a delay obtaining abortion?^*,b^			
Yes	20 (46.5)	4 (18.2)	16 (76.2)
No	23 (53.5)	18 (81.8)	5 (23.8)
Abortion method obtained			
Procedure by a health care provider	24 (63.2)	13 (59.1)	11 (68.8)
Medication/pills prescribed by a health care provider	14 (36.8)	9 (40.9)	5 (31.3)
Type of facility			
Clinic that specializes in abortion services	31 (72.1)	19 (86.4)	12 (57.1)
Other types of facility	12 (27.9)	3 (13.6)	9 (42.9)
Abortion involved virtual contact with provider	12 (27.9)	5 (22.7)	7 (33.4)

^a^ Individual “Yes” responses reported.

^b^ “Yes” responses aggregated.

^*^ *p* < 0.001.

### Abortion

Of the 21 respondents who obtained or sought an abortion during the COVID-19 pandemic, 16 (76.2%) reported experiencing a delay in obtaining abortion care (*i.e.*, COVID-19 and non-COVID-19-related reasons). Almost all (*n* = 14) of those who obtained an abortion and experienced a delay cited the pandemic as at least part of the reason for the delay. These findings compare with reports from 18.2% (*n* = 4) of those who experienced a delay obtaining abortion care before the pandemic (*p* < 0.001), indicating a trend that people were more likely to experience a delay accessing abortion during the pandemic.

More than half of those who had an abortion before the pandemic (59.1%) had a procedure provided by a health care provider (*i.e.*, a surgical or procedural abortion) and 40.9% had a medication abortion. Most of those who obtained an abortion during the pandemic (68.8%) had an abortion procedure by a health care provider, and 31.3% had a medication abortion. In both groups, most respondents sought abortion care at a clinic that specializes in abortion services (such as Planned Parenthood), although a higher percentage of respondents who sought abortion care during the pandemic reported seeking care at a clinic that provides mostly other types of services, a private doctor's office, or a hospital (42.9% during COVID-19 vs. 13.6% before COVID-19).

In addition, we found that a significantly higher proportion of respondents who gave birth or were pregnant during the pandemic considered obtaining an abortion (39.1%) (*i.e.*, COVID-19 and non-COVID-19-related reasons), compared with those who gave birth before the pandemic (7.6%; *p* < 0.001; Table 3). Of those who were pregnant during COVID-19 and considered abortion (*n* = 239), 65.7% reported that the pandemic was at least part of the reason they considered abortion during their pregnancy.

**Table 3.** Consideration of Abortion During Pregnancy

Variables	Total sample (***N*** = 850), ***n*** (%)	Pregnant before COVID-19 and did not have a miscarriage or an abortion (***n*** = 238), ***n*** (%)	Pregnant during COVID-19 and did not have a miscarriage or an abortion (***n*** = 612), ***n*** (%)
Considered abortion during pregnancy?^a^			
Yes, due to COVID-19	133 (15.6)	—	133 (21.7)
Yes, due to COVID-19 plus other reasons	24 (2.8)	—	24 (3.9)
Yes, due to reasons other than COVID-19	100 (11.8)	18 (7.6)	82 (13.4)
No	593 (69.8)	220 (92.4)	373 (60.9)
Considered abortion during pregnancy?^*,b^			
Yes	257 (30.2)	18 (7.6)	239 (39.1)
No	593 (69.8)	220 (92.4)	373 (60.9)

^a^ Individual “Yes” responses reported.

^b^ “Yes” responses aggregated.

^*^ *p* < 0.001.

### Miscarriage

Of the 977 respondents who were pregnant during and before COVID-19, 73 (7.5%) experienced miscarriage: 39 (53.4%) who were pregnant during COVID-19 and 34 (46.6%) who were pregnant before COVID-19 (Table 4).

**Table 4.** Miscarriage-Related Experiences of Those Pregnant Before the COVID-19 Pandemic Compared with Those Pregnant During the Pandemic

Variables	Total sample who experienced a miscarriage, ***n*** (%) (***n*** = 73)	Experienced a miscarriage before COVID-19 (***n*** = 34), ***n*** (%)	Experienced a miscarriage during COVID-19 (***n*** = 39), ***n*** (%)
Delayed obtaining miscarriage care?^a^			
Yes, due to COVID-19	9 (12.3)	—	9 (23.1)
Yes, due to COVID-19 plus other reasons	5 (6.8)	—	5 (12.8)
Yes, due to reasons other than COVID-19	8 (11.0)	5 (14.7)	3 (7.7)
No	41 (56.2)	22 (64.7)	19 (48.7)
Did not seek care	10 (13.7)	7 (20.6)	3 (7.7)
Delayed obtaining miscarriage care?^*,b^			
Yes	22 (30.1)	5 (14.7)	17 (43.6)
No/did not seek care	51 (69.9)	29 (85.3)	22 (56.4)
Health care provider delayed in seeing you for miscarriage care?^a^			
Yes, due to COVID-19	11 (15.1)	—	11 (28.2)
Yes, due to reasons other than COVID-19	5 (6.8)	2 (5.9)	3 (7.7)
No	47 (64.4)	25 (73.5)	22 (56.4)
Did not seek care	10 (13.7)	7 (20.6)	3 (7.7)
Health care provider delayed in seeing you for miscarriage care?^*,b^			
Yes	16 (21.9)	2 (5.9)	14 (35.9)
No/did not seek care	57 (78.1)	32 (94.1)	25 (64.1)
Types of appointments/consultations available from health care provider for miscarriage			
Regular in-person appointments	53 (77.9)	29 (90.6)	24 (66.7)
Virtual (online) appointments	36 (57.1)	12 (44.4)	24 (66.7)
Phone appointments	52 (78.8)	20 (66.7)	32 (88.9)
Online message portal for questions	36 (60.0)	13 (48.1)	23 (69.7)

^a^ All individual responses reported.

^b^ All “Yes” and “No/Did not seek care” responses aggregated, respectively.

^*^ *p* < 0.01.

Of the 39 respondents who had miscarriages during the pandemic, 17 (43.6%) reported delaying miscarriage care, compared with 5 (14.7%) of those who had a miscarriage before the pandemic (*p* < 0.01) (aggregating all “Yes” and “No/Did not seek care” responses, respectively). More than a third (35.9%) of the 39 respondents who miscarried during the pandemic reported delaying care because of the pandemic; more than a quarter (28.2%) of those who miscarried during the pandemic reported that their health care provider delayed seeing them for miscarriage care due to COVID-19. Compared with those who miscarried before the pandemic, a significantly higher proportion of those who miscarried during the pandemic reported that their health care provider delayed seeing them for any reason (5.9% vs. 35.9%, respectively; *p* < 0.01).

More participants who miscarried before COVID-19 reported having regular in-person appointments available to them for care from their health care provider compared with those who miscarried during COVID-19 (90.6% vs. 66.7%). Conversely, more participants who miscarried during COVID-19 reported having phone appointment services available from their health care provider for care, compared with those who miscarried before COVID-19 (88.9% vs. 66.7%).

### Open-ended comments about miscarriage experience

Of the 73 respondents who had a miscarriage before or during COVID-19, 59 (80.8%) participants provided a valid response to the open-ended question about their experience (28 who miscarried before the pandemic and 31 during the pandemic). We aggregated the responses into two main themes: received social support or had positive experiences with health care and did not receive social support or had negative experiences with health care (Table 5). Several participants (*n* = 6) who sought miscarriage care during COVID-19 mentioned the direct impact of the pandemic on the safety and quality of the care they received.

**Table 5.** Open-Ended Comments About Miscarriage

Categories	Experienced a miscarriage before COVID-19 (***n*** = 28)	Experienced a miscarriage during COVID-19 (***n*** = 31)
Positive/neutral Received social support; positive or neutral health care experience	12	11
Negative Did not receive social support; negative health care experiences	14	17
Other Did not want to discuss; did not seek medical care	2	3

One participant wrote “…I refused to go to [the] hospital because of the COVID-19.” Another participant explained, “My personal [doctor's] office was closed, and I was only able to talk to my doctor. I was also unable to go to the hospital out of fear of contracting COVID-19.” This statement is illustrative of the finding that health care providers were more accessible by phone than in person during the pandemic, and that respondents delayed or avoided seeking miscarriage care because of COVID-19 concerns.

Other respondents expressed that they faced challenges with their medical providers when they sought miscarriage care during the pandemic. One participant wrote, “It was hard because the doctors were unsure how to handle the situation. It was at the beginning of [the] COVID outbreak…” Another wrote, “… Due to COVID-19, I feel that the doctors were not as compassionate as they normally would have been. They were more leery and afraid.”

## Discussion

Those who sought abortion or miscarriage care during the COVID-19 pandemic experienced more delays in getting care than those who sought services before the pandemic. These delays took place despite NYS's expansive abortion legislation, including the Reproductive Health Act that was enacted in 2019.^32^ Our findings suggest a gap between policy intentions to make abortion widely accessible and the reality of access during a global pandemic, as some patients were hesitant to access care and some health care providers delayed providing care.

Surprisingly, a higher proportion of respondents who obtained abortions during the pandemic had procedural abortions compared with those who obtained abortions before the pandemic (68.8% vs. 59.1%). This may have been because of confusion or concern about access to abortion, with knowledge that medication abortion is a longer process than procedural abortion. Preferences for type of abortion are starting to be more closely examined in the literature.^33^

Our findings also show that significantly more people who were pregnant during the pandemic considered abortion, compared with those pregnant before the pandemic (39.1% vs. 7.6%). The purpose of this question was to obtain a general sense of possible differences in the number of individuals considering abortion based on whether the pregnancy was before or during the pandemic. While the reason why individuals may consider obtaining an abortion is also an important question, it was outside the scope of this study. These findings align with other evidence that suggests that the pandemic affected reproductive decision-making, further supporting the need for access to all pregnancy outcome options.^34–36^

While the abortion policy in NYS is expansive, it can be made more expansive in practice by increasing access to medication abortion, especially in light of the *Dobbs* decision and recent judicial challenges to mifepristone.^37^ The National Academies of Science, Engineering, and Medicine concluded in 2018 that there is no medical need for abortion medications to be administered in the physical presence of a health care provider.^38^ International organizations have been mailing abortion medications to patients for the last 15 years, while state abortion restrictions and demands have led to an influx of U.S.-based organizations providing abortion medication to those living where abortion is inaccessible or criminalized.^39–41^ This involves “no-test” abortions, a type of medication abortion that does not require any physical interaction between the provider and patient, reducing the risk of COVID-19 transmission or legal surveillance.^42^

Increased access to telehealth for abortion is important in the context of *Dobbs*, which has led to abortion bans and extensive restrictions across the United States. Access to medication abortion via telehealth may help those who are unable to access abortion in their home states, as well as clinics experiencing surges in patient volume in states where abortion access is protected.^43–45^

Telehealth medication abortion alone will not meet the needs of all patients seeking abortions or miscarriage care. As our findings show, the majority of those who obtained an abortion before and during the pandemic did so at an in-clinic procedure. Some patients may be unsure of how to navigate telemedicine visits or lack a private place to speak candidly about their abortion or miscarriage care needs. Some patients may prefer to meet with a provider in person. After a certain point in pregnancy, abortion and miscarriage care require procedural intervention. Some patients may not have access to reliable internet, as digital access differs across communities and neighborhoods.^46^

However, if abortion and miscarriage care are more accessible through telehealth, health care providers and clinics may be less burdened by in-person appointments and better able to serve patients seeking procedural abortions. In addition, having more abortion and miscarriage management options gives patients more control over their reproductive needs.

Abortion and miscarriage care training for different kinds of medical providers, including advanced practice clinicians and nonspecialists, is important for increasing access to these services.^47^ Our findings support training nonspecialist providers, as almost 30% of those who sought abortions did so at a facility that did not specialize in abortion.

## Limitations

There were several limitations in our study. The sampling frame was derived from a Qualtrics panel and, therefore, not statistically representative of the NYS population. The sample sizes for respondents who experienced miscarriage or were seeking abortions were relatively small, which limited the analyses we could perform, such as stratification by race, ethnicity, and socioeconomic status. Abortions may have been underreported in the sample given the well-established evidence base of underreport in surveys.^48^ Data on miscarriage may also be underreported due to the reluctance of some participants to provide information about this personal, oftentimes devastating, experience.

Recall bias may have also been an issue, especially for those who were pregnant before the pandemic as that experience was longer ago. In addition, respondents who delayed care were asked to provide a reason why they delayed care, which may have discouraged reporting. Respondents who were pregnant and considered abortion before the pandemic were not asked why they considered abortion. Both abortion and miscarriage remain highly stigmatized in the United States, making them challenging topics to study.

## Implications for Practice and Policy

Abortion and miscarriage care are essential health care services that must be fully available during pandemics or other public health emergencies.^49–51^ We know that delays in accessing these services can increase costs and risks for pregnant people.^10,52,53^ Our findings suggest that special efforts need to be used now to ensure access to abortion and miscarriage care during future public health crises. Telehealth for abortion services and miscarriage care should be expanded throughout the United States, such as ending state requirements for abortion medications to be dispensed in person or that the prescribing physician be present for the first medication.^54^ There is also evidence that preabortion ultrasounds are unnecessary in the first 12 weeks of pregnancy, and the elimination of this requirement could also expand access to abortion.^55^

The implications of these findings are exacerbated by the ending of the constitutional right to abortion resulting from the *Dobbs* decision. Specifically, the Society of Family Planning reports that between June 2022 and June 2023, when the *Dobbs* decision was made, states with total abortion bans had 94,930 fewer clinician-provided abortions. This represents a 100% decrease in the number of abortions provided pre-*Dobbs*.^56^ These data show that needs for abortion are not being met. One important note on these data is that self-managed abortions cannot be accurately counted.

## Conclusion

Among respondents in our sample, those who sought abortion or miscarriage care during the COVID-19 pandemic experienced more delays in getting care than those who sought services before the pandemic. Access to abortion and miscarriage care is key to people having control over their reproductive lives.^57,58^ Pandemics and other public health emergencies can exacerbate existing vulnerabilities, making access to abortion and miscarriage care more difficult. Access to miscarriage and abortion care are time-sensitive health care needs, and delays to either can be dangerous.^59^ Efforts to expand abortion and miscarriage care should be focused on both restoring legal rights and ensuring services are accessible for all.

## Abbreviations Used

NYS: New York State
SRH: sexual and reproductive health
